# Analysis of artery injury types and clinical characteristics of patients with transcatheter angioembolization after percutaneous nephrolithotomy

**DOI:** 10.3389/fsurg.2024.1429821

**Published:** 2025-01-13

**Authors:** Xianghu Meng, Rong Cong, Yibo Hua, Zengjun Wang, Ninghong Song, Wei Yang, Rijin Song

**Affiliations:** ^1^ Department of Urology, First Affiliated Hospital with Nanjing Medical University, Nanjing, China; ^2^ Department of Interventional Radiology, First Affiliated Hospital with Nanjing Medical University, Nanjing, China

**Keywords:** percutaneous nephrolithotomy, transcatheter angioembolization, bleeding, tract, pseudoaneurysm, arteriovenous fistula

## Abstract

**Background:**

There is no systematic classification of renal vascular injuries conducted for severe post-percutaneous nephrolithotomy (PCNL) bleeding.

**Aim:**

The aim of the present study was to explore the various types of artery injury and clinical characteristics of patients who underwent transcatheter angioembolization (TAE) after PCNL.

**Methods:**

A retrospective analysis was performed on 52 patients who underwent renal arteriography (RA) because of severe bleeding after PCNL between April 2009 and December 2023. Among the patients, 38 underwent TAE due to positive RA results. Clinical data on the TAE patients, such as gender, age, body mass index, TAE interval, hemoglobin (Hb) decrease, operation time, stone size, the number and size of tracts, and clinical bleeding type, were summarized. The types of artery injury in TAE patients and their relationships with clinical characteristics were analyzed.

**Results:**

Retrospective analysis revealed that, among the 38 TAE patients (32 males and 6 females), the mean TAE interval, average Hb decrease, mean tract number, and mean tract size reached 5.00 (6.25) days, 44.50 (24.50) g/L, 1 (0.25), and F20(6), respectively. Among the TAE patients, four kinds of vascular injury were observed, namely, 18 cases of pseudoaneurysm (PA), 12 cases of arteriocaliceal fistula (ACF), 7 cases of arteriovenous fistula (AVF), and 1 case of arterioperirenal fistula (APF). Analysis of the clinical characteristics of the three types of vascular injury (PA, ACF, and AVF) revealed that the number of tracts was the only factor that differed.

**Conclusion:**

The RA results indicate that the types of postoperative renal artery injury mainly include PA, ACF, AVF, and APF, and the number of tracts may be related to the type of vascular injury.

## Introduction

Urologists have accumulated experience in percutaneous nephrolithotomy (PCNL), and PCNL equipment has undergone vast development. Accordingly, the application of PCNL technology, which has become the preferred treatment for kidney stones ≥2 cm or complex urinary stones, has spread internationally (1–3). The PCNL process primarily comprises three operative steps, namely, puncture, tract dilation, and stone fragmentation. Despite being a minimally invasive surgical procedure, renal vessel damage during any of these steps can result in postoperative bleeding, which is the most prevalent complication of PCNL. It can usually be controlled via conservative methods (4).

For patients who encounter severe postoperative bleeding after PCNL and do not respond to conservative treatments, transcatheter angioembolization (TAE) stands as an effective and safe alternative with the success rate as high as 90%–100% (5–8). Patients who suffer from severe postoperative bleeding after PCNL often experience arterial injury. However, thus far, no systematic classification of renal vascular injuries has been conducted. Li et al. classified post-PCNL renal arterial injuries into three types: pseudoaneurysm (PA), arteriovenous fistula (AVF), and arterial lacerations (6). Choi et al. categorized arterial injuries as extravasation, PA, and AVF (7). Yang et al. classified post-PCNL vascular injuries as PA, AVF, and bleeding spot (8). Venkateswarlu et al. classified postoperative bleeding as PA, AVF, or active bleeding (9). Kervancioglu et al. divided postoperative bleeding into PA, AVF, arteriocaliceal fistula (ACF) (10). This retrospective study analyzed the clinical characteristics of TAE patients in our center over a 14 year period, systematically analyzed the types of vascular injuries, and compared the differences in the clinical characteristics of various injury types.

## Methods

### Study population and design

**Figure 1:**
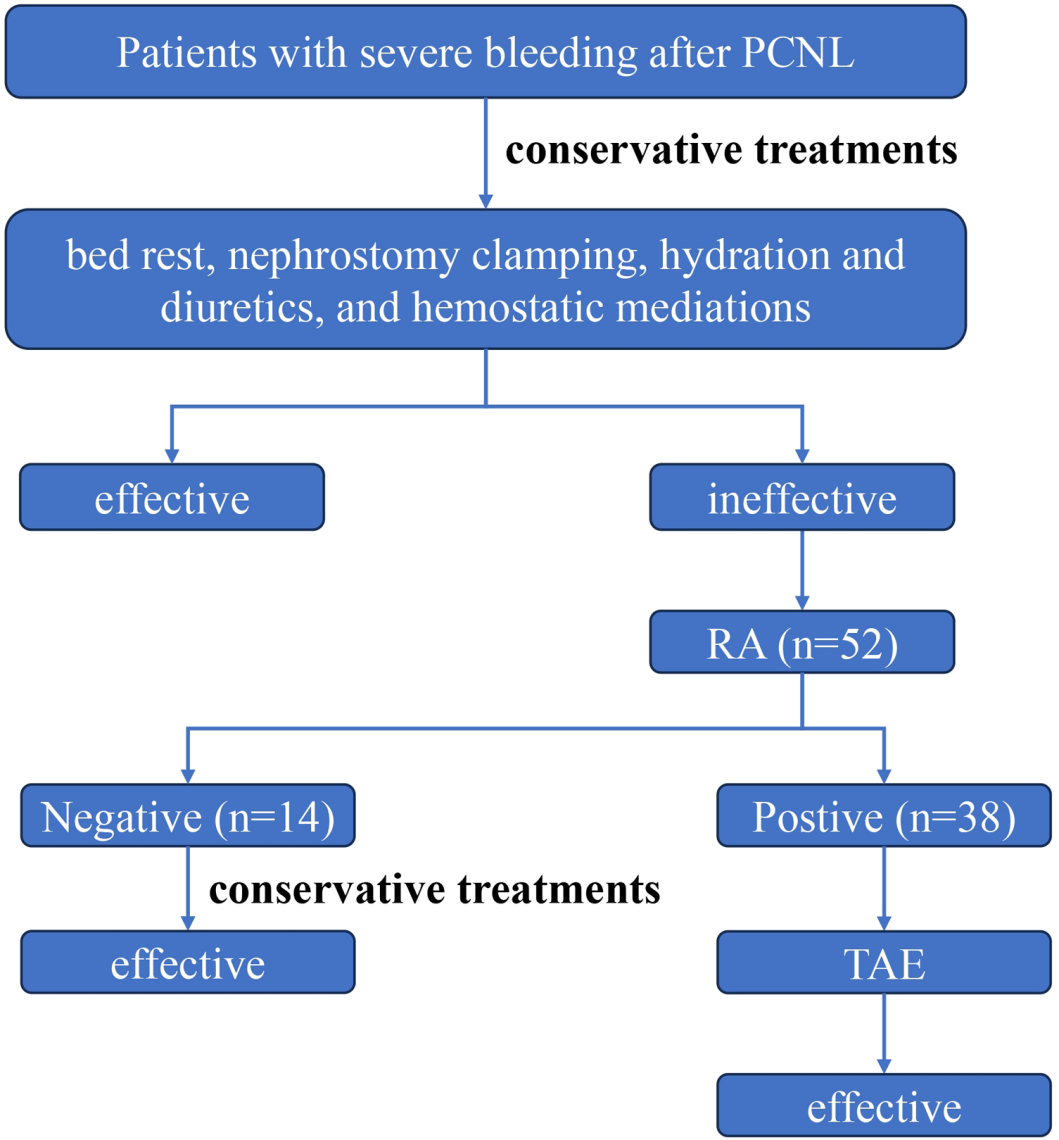
The clinical management flowchart for patients with severe bleeding after PCNL. Patients with severe bleeding after PCNL are initially treated by conservative measures. If conservative treatment is ineffective, a RA examination is recommended. If the RA is negative, conservative management continues; if the RA is positive, the patient undergoes TAE treatment.

A retrospective analysis was conducted on 52 patients who underwent renal arteriography (RA) examination after severe postoperative bleeding following PCNL from April 2009 to December 2023. Among these patients, 38 had positive RA findings and underwent TAE. The specific clinical processing procedure is shown in Figure 1. Clinical data, including gender, age, BMI, TAE interval, hemoglobin (Hb) decrease, bleeding type, preoperative urine routine and urine culture results, preoperative hydronephrosis (11), operation time, stone size, preoperative creatinine (Cr) level, number of tracts, tract size, and baseline diseases, were summarized. The types of vascular injuries in these patients were analyzed, and the relationships between vascular injury types and clinical characteristics were summarized. Inclusion criterion was ① all patients who underwent RA examination after PCNL. Exclusion criteria were ① patients with negative RA findings; and ② patients lacking follow-up data after surgery.

### Procedure

RA and TAE are common procedures done for diagnosis and treatment of renal hemorrhage, and thus, only a brief summary is provided in this article. Patients with severe postoperative bleeding after the implementation of failed conservative methods, including bed rest, diuretics and hydration, nephrostomy clamping, and hemostatic mediations, were subjected to RA examination. The procedure was accomplished by interventional radiologists using digital subtraction angiography equipment. Local anesthesia was administered, and percutaneous vascular access was established in the right or left femoral artery. The Seldinger technique was used to position a 4–5 French guiding sheath introducer. The renal artery was accessed via renal angiography with the use of a Cobra catheter. The type, size, and location of renal vascular injury were determined through selective arterial angiography. TAE was conducted based on the findings of RA examination. In the case of negative RA findings, patients returned to the ward for continued conservative treatment. Positive RA findings prompted transcatheter embolization, which was performed using platinum microcoils, N-butyl cyanoacrylate, particles, or Spongostan, depending on the type and severity of vascular injury. Embolization was followed by repeat angiography to confirm hemostasis and exclude complications.

### Observation indicators

The following data on TAE patients were recorded and analyzed: gender, age, BMI, TAE interval, Hb decrease, bleeding type, preoperative urine routine and urine culture results, preoperative hydronephrosis (11), operation time, stone size, preoperative Cr level, number of tracts, tract size, and baseline diseases (hypertension, diabetes, history of previous ipsilateral PCNL or ureterotomy, and renal insufficiency). The operation time spanned from ureteral catheter placement to suturing and fixation of the nephrostomy tube or suturing of the percutaneous renal tract.

Based on the literature, bleeding types were classified as follows: sudden onset, intermittent, and continuous slow (6, 12). Given the lack of specific definitions for these classifications, the following conditions were considered: sudden onset bleeding type refers to the sudden drop in Hb levels after surgery or bladder obstruction due to sudden bleeding or continuous drainage of bright-red blood from the nephrostomy tube; intermittent bleeding type refers to intermittent gross hematuria and an intermittent decrease in Hb levels; and continuous slow bleeding indicates the persistent drainage of dark red or pale red blood in urine or from the nephrostomy tube, with a slow, continuous decrease in Hb levels.

### Statistical analysis

Statistical analysis was accomplished using SPSS 20.0. As the number of included patients is relatively low, continuous data are presented as median (interquartile range) and were analyzed via Kruskal–Wallis H(K) test. *post hoc* test for pairwise comparisons was adjusted by Bonferroni method. Categorical data are presented as percentages and were analyzed using chi-square test. Statistical significance was considered at a *p*-value less than 0.05.

## Results

### General clinical data of patients

Table 1 summarizes the clinical characteristics of the 38 TAE patients included in the study. After TAE treatment, bleeding was successfully controlled in all patients. These patients included 32 males and 6 females with a mean age of 51.50 (18.25) years, mean BMI of 24.05 (5.06) kg/m^2^, mean TAE interval of 5.00 (6.25) days, average Hb decrease of 44.50 (24.50) g/L, average operation time of 90.50 (54.50) min, mean stone size of 41.00 (39.50) mm, preoperative urine routine white blood cell count of 158 (840), Cr level of 77.55 (24.15) μmol/L, mean tract number of 1 (0.25), and tract size of F20(6) (Table 1). According to the different clinical bleeding types, continuous slow bleeding was the main bleeding type among the TAE patients (19 patients), followed by sudden onset (15 patients), and intermittent (4 patients) bleeding.

**Table 1:** Clinical characteristics of TAE patients.

Patients (*n*)	38
Sex (*n*)	
Male	32
Female	6
Age (years)	51.50 (18.25)
BMI (kg/m^2^)	24.05 (5.06)
Preoperative urine leukocyte counts	158 (840)
Preoperative urine culture	
Positive	12
Negative	26
Operation time (min)	90.50 (54.50)
Stone size (mm)	41.00 (39.50)
Preoperative creatinine (μmol/L)	77.55 (24.15)
Degree of hydronephrosis	
None	12
Mild	15
Moderate	8
Severe	3
Preoperative basic diseases	
Hypertension (Yes/No)	10/28
Diabetes (Yes/No)	8/30
Past ipsilateral urolithiasis surgery (Yes/No)	8/30
Renal insufficiency (Yes/No)	3/35
No. of tracts	1 (0.25)
Size of tract	20 (6)
TAE interval (days)	5.00 (6.25)
Hb decrease (g/L)	44.50 (24.50)
Kinds of artery injury	
PA	18
ACF	12
AVF	7
APF	1
Bleeding type	
Sudden onset	15
Intermittent	4
Continuous slow	19

TAE, transcatheter angioembolization; PA, pseudoaneurysm; ACF, arteriocaliceal fistula; AVF, arteriovenous fistula; APF, arterioperirenal fistula.

The TAE interval ranged from the first post-PCNL day to the day of TAE. Hb decrease referred to the decrease in Hb from post-PCNL day to the day of RA. Based on the equivalence of one unit of red blood cells or whole blood to 1 g/dl Hb, the changes in Hb were corrected for patients who received blood transfusion prior to TAE (6).

### Classification and characteristics of artery injury patients after PCNL

**Figure 2:**
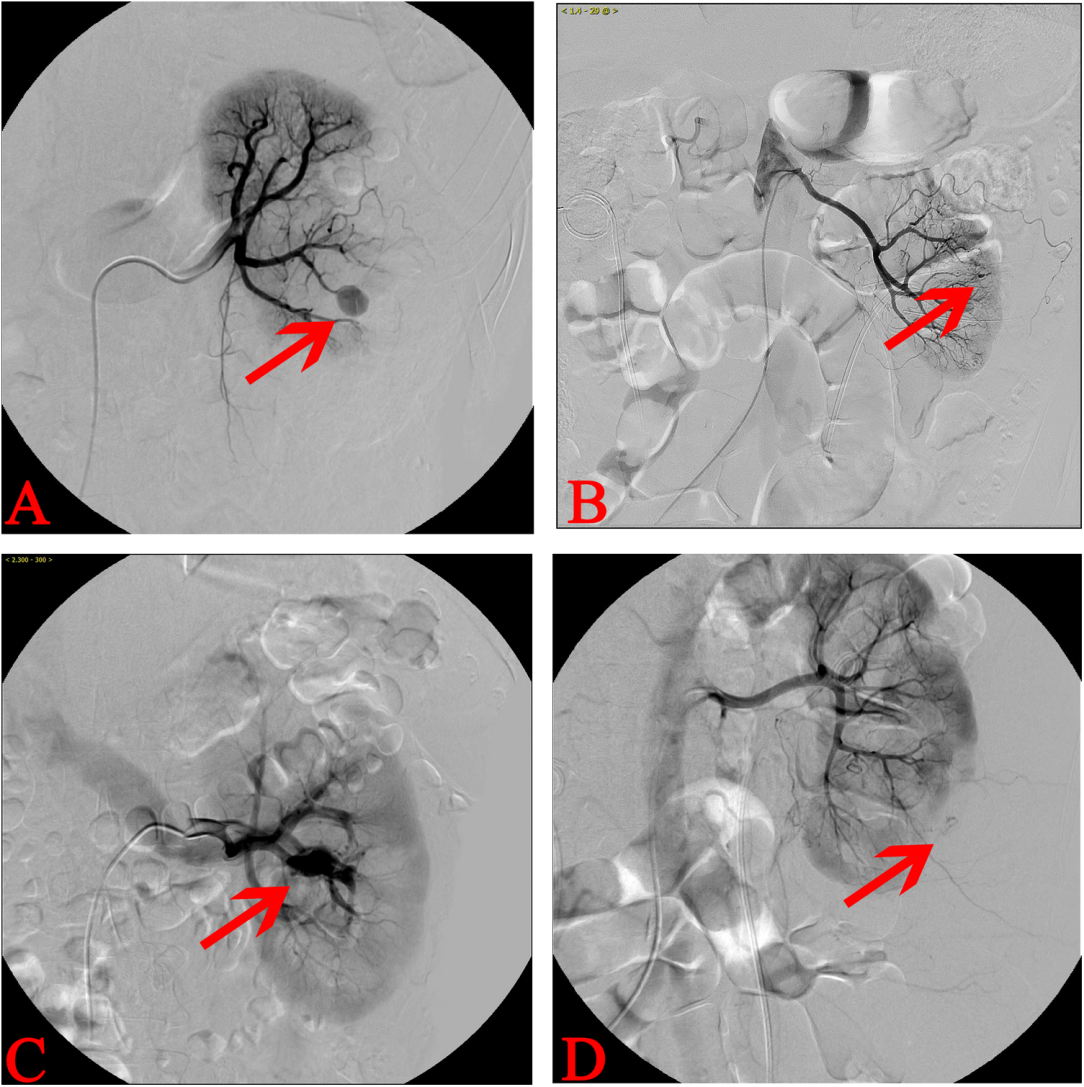
Different types of artery injury after PCNL. **(A)** Left renal RA suggesting that PA is the most common type of vascular injury after PCNL. **(B)** Left RA showing the extravasation of contrast agent and indicating vascular injury. The patient had severe hematuria and was clinically classified as ACF. **(C)** RA revealing the contrast agents found in the renal veins early during angiography, which implies AVF. **(D)** Left kidney RA revealing the extravasation of contrast agent to the perirenal area with the inferior kidney compressed. The patients often showed decreased Hb with no evident gross hematuria, and imageological examinations usually suggest perirenal hematoma. This type of artery injury was APF.

The types of vascular injuries among the 38 TAE patients were classified into four categories based on angiographic images and clinical bleeding characteristics: 18 cases of PA (Figure 2A), 12 cases of ACF (Figure 2B), 7 cases of AVF (Figure 2C), and 1 case of arterioperirenal fistula (APF) (Figure 2D). Given that only one patient with APF was included in the study, this patient was excluded from the statistical analysis. Only the clinical characteristics of patients with PA, ACF, and AVF were analyzed. Continuous data including age, BMI, urine leukocyte counts, stone size, Cr, TAE interval, Hb decrease, operation time, No. of tracts, and size of tract were analyzed via Kruskal–Wallis H(K) test. Categorical data including gender, urine culture, hydronephrosis, hypertension, diabetes, previous surgery, renal insufficiency, and bleeding type were analyzed using chi-square test. No statistically significant differences were observed among the three groups of patients in terms of gender, age, BMI, TAE interval, Hb decrease, operation time, stone size, preoperative urine routine white blood cell count, Cr level, and baseline disease status, such as hypertension, diabetes, history of previous surgeries, or renal insufficiency. The only parameter showing a statistically significant difference was the number of tracts (*P* = 0.032) (Table 2). And the *post hoc* test for pairwise comparisons of the number of tracts indicated the ACF group had more tracts than AVF group (Table 2).

**Table 2:** The comparison of clinical data among different kinds of artery injury patients treated with TAE.

Clinical data		TAE patients			*P* value	APF
		PA	ACF	AVF		
Gender	Male	15	10	6	1.00^a^	1
	Female	3	2	1		
Age (y)		51.50 (16.00)	52.50 (24.50)	47.00 (19.00)	0.65^b^	70
BMI (kg/m2)		23.86 (7.17)	24.96 (5.25)	22.48 (3.16)	0.49^b^	21.26
Urine leukocyte counts		158.50 (812.50)	190.00 (858.00)	37.00 (338.00)	0.21^b^	5,220
Urine culture	Positive	7	3	2	0.82^a^	
	Negative	11	9	5		1
Stone size (mm）	37.50 (40.00)	51.30 (43.25)	35.00 (18.00)	0.22^b^	100	
Cr (μmol/L)	77.55 (20.25)	73.05 (37.90)	87.00 (30.6)	0.50^b^	107	
Hydronephrosis	None	7	3	2	0.64^a^	1
	Mild	8	4	3		
	Moderate	3	3	1		
	Severe	0	2	1		
Hypertension	Yes	5	5	0	0.15^a^	
	No	13	7	7		1
Diabetes	Yes	4	2	2	0.88^a^	
	No	14	10	5		1
Previous surgery	Yes	4	3	1	1.00^a^	
	No	14	9	6		1
Renal insufficiency	Yes	2	1	0	1.00^a^	
	No	16	11	7		1
TAE interval (d)		5.50 (7.50)	4.00 (6.75)	6.00 (3.00)	0.25^b^	4
Hb decrease (g/L)		46.00 (27.00)	34.00 (22.50)	46.00 (10.50)	0.10^b^	50
Operation time (min)		93.50 (47.00)	101.50 (80.50)	80.00 (43.00)	0.88^b^	180
No. of tracts		1 (0)	1.5 (1)*	1 (0)	0.03^a^	1
Size of tract		20 (6)	18 (4)	22 (6)	0.20^a^	24
Bleeding type	Sudden onset	9	4	2	0.49^a^	
	Intermittent	3	1	0		
	Continuous slow	6	7	5		1

TAE, transcatheter angioembolization; PA, pseudoaneurysm; ACF, arteriocaliceal fistula; AVF, arteriovenous fistula; APF, arterioperirenal fistula.

*P* value: Considering that the study included a single participant with APF, this individual was omitted from the statistical analysis to ensure the integrity and reliability of the results. Consequently, the analysis was focused solely on the clinical characteristics of patients diagnosed with PA, ACF, and AVF.

^a^ Chi-suqare test.

^b^ Kruskal–Wallis H(K) test.

*P < 0.05, compared with AVF group.

## Discussion

Retrospective analysis of 14 years of single-center data showed that TAE effectively controlled all severe postoperative bleeding after PCNL of patients who received ineffective conservative treatments, with a mean postoperative TAE interval of 5.00 (6.25) days and a mean Hb decrease of 44.50 (24.50) g/L. All patients requiring TAE after PCNL suffered from arterial injuries. Previous studies lacked a standardized classification for post-PCNL vascular injuries. Past reports also described other types of injuries, namely, PA, AVF, arterial lacerations, extravasation, bleeding spot, active bleeding, and ACF, with PA and AVF being the most commonly mentioned (6–10).

In this study, we classified the types of vascular injuries into PA, ACF, AVF, and APF (Figure 2). PA is defined as a vascular injury where surrounding connective tissue forms an imaging characteristic similar to an aneurysm (6, 13, 14). AVF occurs when the artery and vein are involved in the injury, which leads to early arterial blood entering the vein (6, 13, 14). ACF refers to an arterial injury connected to the collecting system (10). APF includes an arterial injury connected to the renal capsule. This type of injury has not been previously reported but may be associated with compression of the renal capsule following bleeding. The patients typically present with a decrease in Hb level, accompanied with back pain and a relatively clear urine color. In addition, imaging examinations often reveal massive retroperitoneal hematoma.

In this study, the types of bleeding included 18 cases of PA, 12 cases of ACF, 7 cases of AVF, and 1 case of APF. A retrospective analysis by Li et al. revealed that among 137 patients with positive RA findings, PA, AVF, and arterial laceration accounted for 47.9%, 19.4%, and 16%, respectively (6). The proportions of PA and AVF in our study were consistent with those reported in previous research, suggesting that PA is the main type of vascular injury after PCNL.

**Figure 3:**
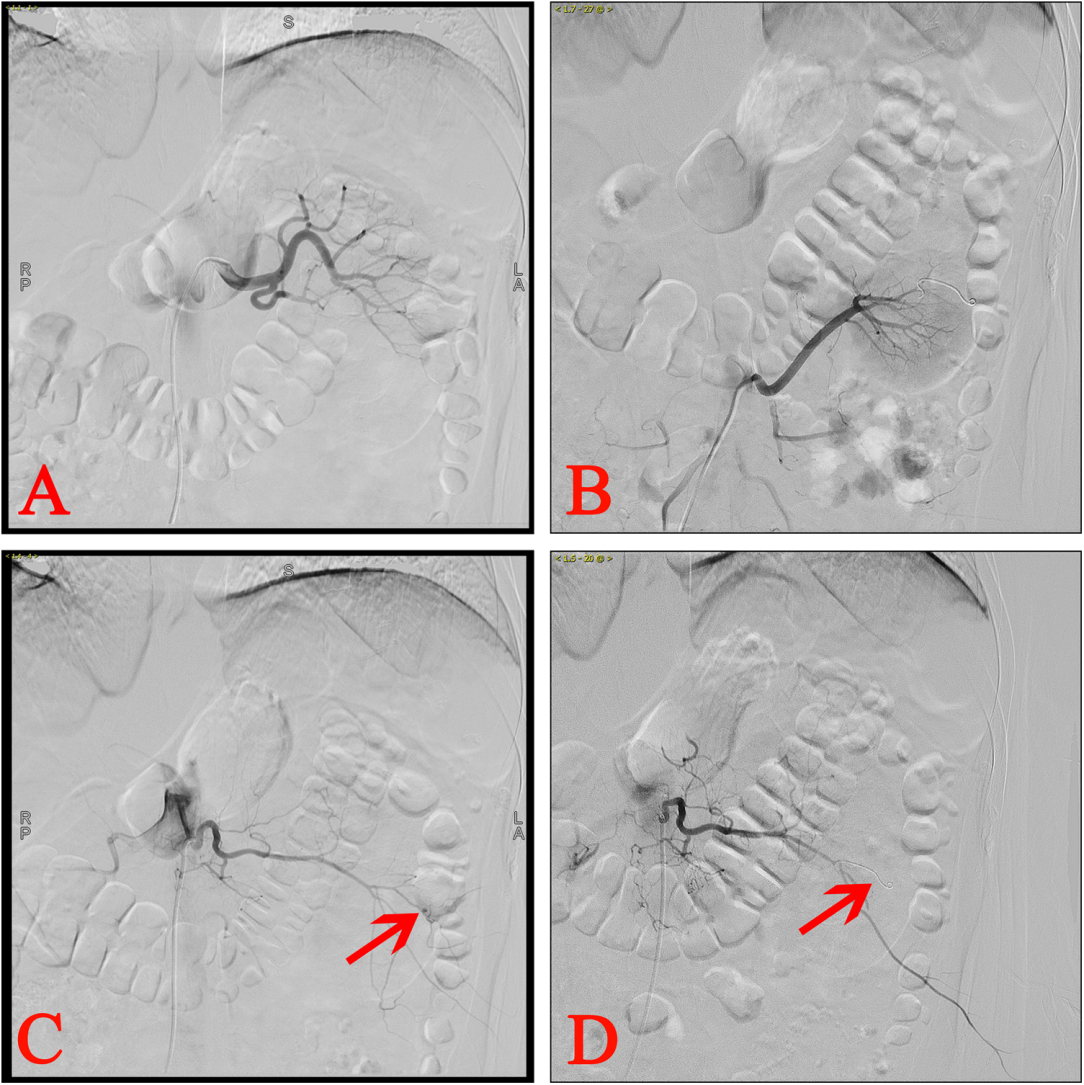
Ra of a patient with lumbar artery injury after PCNL. **(A)** Left RA showing no abnormalities. **(B)** Left accessory RA revealing no abnormalities. **(C)** Lumbar arteriography suggested PA. **(D)** The PA was successfully embolized.

Performing RA on patients with severe postoperative bleeding after PCNL did not necessarily indicate that the renal artery was the sole site of injury. Postoperative bleeding may result from damage of any artery during PCNL access or stone fragmentation. Figure 3 illustrates a patient with RA positivity who experienced severe bleeding after PCNL in this study. The patient had intermittent postoperative bleeding, and conservative treatment was ineffective, leading to the RA examination. However, RA examination revealed no abnormalities in the renal artery or accessory renal arteries but detected PA in the lumbar artery. The bleeding ceased following embolization. Consequently, when performing an RA examination, it is important to consider not only the renal artery but also potential injuries to the accessory renal arteries, lumbar arteries, renal capsular artery, and intercostal arteries (7, 15–17).

The type of clinical bleeding detected in patients with TAE remains controversial. Li et al. retrospectively observed intermittent bleeding (46.5%) as the main type of TAE (6). The retrospective research of Ran et al. revealed sudden onset bleeding (52.9%) as the main type (12), whereas in the present study, continuous slow bleeding (50%) was the main type. Hence, to further gain insights into the clinical characteristics of patients with various types of vascular injuries, we conducted a comparative analysis of their clinical characteristics to provide guidance for interventional physicians during TAE. In this retrospective analysis, the types of vascular injuries were classified into four categories. Subgroup analysis (PA, ACF, and AVF) revealed no statistically significant differences in patient age, BMI, operation time, stone size, preoperative baseline disease status, bleeding type, etc. However, statistically significant difference was observed only in the number of tracts, suggesting that this parameter may be a predictor of different vascular injuries in TAE patients.

Currently, there is no research discussing the types of renal artery injuries in patients with severe bleeding after PCNL. However, regardless of the different type of vascular injury, TAE has been proven to be an effective method for treating severe bleeding after PCNL (6, 7, 9, 10, 13). Understanding different types of vascular injuries may assist interventional radiologists in performing RA examinations and in selecting embolic materials during TAE. In addition, although this study found that the number of tracts might be a factor in predicting the type of vascular injury, the mechanism between the two has not been clarified and requires further research.

Limitations of this study: The retrospective design of this study may introduce potential bias in the results. Conducting a prospective randomized controlled trial for patients with severe postoperative bleeding following PCNL is challenging due to the significant psychological burden it places on both surgeons and patients. Additionally, the study included only one case of APF, which was insufficient for statistical analysis despite its notable clinical characteristics. Further research with larger sample sizes is needed to enhance the validity of these findings.

## Conclusion

This retrospective analysis revealed that TAE effectively controlled severe postoperative bleeding after PCNL. The types of vascular injuries in TAE patients were classified as PA, ACF, AVF, or APF. Further analysis revealed the number of tracts as a possible predictor of the different types of vascular injuries in TAE patients.

## Data Availability

The raw data supporting the conclusions of this article will be made available by the authors, without undue reservation.
